# Long-Term Outcomes in Two-Year Follow-Up after Primary Treatment in Patients with a Prior Venous Thromboembolic Event: A Prospective, Observational, Real-Life Study

**DOI:** 10.3390/jcm13051343

**Published:** 2024-02-27

**Authors:** Gualtiero Palareti, Emilia Antonucci, Eugenio Bucherini, Antonella Caronna, Antonio Chistolini, Angela Di Giorgio, Rosella Di Giulio, Anna Falanga, Vittorio Fregoni, Mariagrazia Garzia, Daniela Mastroiacovo, Marco Marzolo, Roberta Pancani, Daniele Pastori, Gian Marco Podda, Anna Maria Rigoni, Luigi Ria, Piera Sivera, Sophie Testa, Adriana Visonà, Roberto Parisi, Daniela Poli, on behalf of the START POST VTE Investigators

**Affiliations:** 1 Fondazione Arianna Anticoagulazione, 40138 Bologna, Italy; e.antonucci@fondazionearianna.org; 2 SS Medicina Vascolare e Angiologia, AUSL Romagna, 48121 Ravenna, Italy; bucherini@gmail.com; 3 Centro per la Diagnosi e la Sorveglianza della Malattia Tromboembolica, UO Medicina Interna D’urgenza, Azienda Ospedaliero Universitaria Policlinico di Modena, Ospedale Civile Baggiovara, 41124 Modena, Italy; caronna.antonella@aou.mo.it; 4 Dipartimento di Medicina Traslazionale e di Precisione Sapienza Università di Roma, 00197 Roma, Italy; antonio.chistolini@uniroma1.it; 5 Angiologia Diagnostica Vascolare Non Invasiva, Fondazione Policlinico Universitario A. Gemelli IRCCS, Università Cattolica del Sacro Cuore, 00168 Roma, Italy; angela.digiorgio@policlinicogemelli.it; 6 U.O. Programma Dipartimentale di Ecografia-AUSL, 40133 Bologna, Italy; rosella.digiulio@ausl.bologna.it; 7 School of Medicine, Università di Milano Bicocca, 20126 Milano, Italy; annafalanga@yahoo.com; 8 Immunoematologia e Medicina Trasfusionale ASST Papa Giovanni XXIII, 24127 Bergamo, Italy; 9 U.O.C. Medicina Generale, Ospedale di Sondalo, ASST della Valtellina e dell’Alto Lario, 23035 Sondalo, Italy; vittorio.fregoni@asst-val.it; 10 UOC Ematologia-Trapianto Cellule Staminali, Azienda Ospedaliera S.Camillo-Forlanini, 00152 Roma, Italy; mgarzia@scamilloforlanini.rm.it; 11 UOSD Angiologia e Diagnostica Vascolare, Ospedale SS Filippo e Nicola, Avezzano (L’Aquila), 67051 L’Aquila, Italy; daniela.mastroiacovo@gmail.com; 12 UOS Angiologia Medica, Ospedale di Rovigo, 45100 Rovigo, Italy; marco.marzolo@aulss5.veneto.it; 13 U.O. Pneumologia, Dipartimento Cardiotoraco-Vascolare, Azienda Ospedaliero-Universitaria Pisana, Ospedale di Cisanello, 56126 Pisa, Italy; roberta.pancani@ao-pisa.toscana.it; 14 Department of Clinical Internal, Anesthesiologic and Cardiovascular Sciences, Sapienza University of Rome, 00197 Roma, Italy; daniele.pastori@uniroma1.it; 15 Medicina Generale 2, ASST Santi Paolo e Carlo, Università degli Studi di Milano, 20142 Milano, Italy; gianmarco.podda@asst-santipaolocarlo.it; 16 UOC di Angiologia, Azienda Ospedaliero Universitaria Integrata, 37126 Verona, Italy; annamaria.rigoni@aovr.veneto.it; 17 UO Medicina Interna, Presidio Ospedaliero di Gallipoli (Lecce), 73014, Gallipoli, Italy; ria.luigi@alice.it; 18 S.C.D.U. EMATOLOGIA Azienda Ospedaliera Ordine Mauriziano, 10128 Torino, Italy; psivera@mauriziano.it; 19 Centro Emostasi e Trombosi, ASST Cremona, 26100 Cremona, Italy; sophie.testa@assst-cremona.it; 20 UOC Angiologia, Dipartimento di Medicina Clinica, Azienda ULSS 2 Marca Trevigiana, Ospedale San Giacomo Apostolo, 31033 Castelfranco Veneto, Italy; adrianavisona@gmail.com; 21 UOSD Ipertensione e Patologie Endocrine Metaboliche Angiologiche, Ospedale SS. Giovanni e Paolo, 30122 Venezia, Italy; r.parisi271158@gmail.com; 22 SOD Malattie Aterotrombotiche, Azienda Ospedaliero Universitaria-Careggi, 50134 Firenze, Italy; polida@aou-careggi.toscana.it

**Keywords:** venous thromboembolism, anticoagulant treatment, bleeding, long-term follow-up, recurrence

## Abstract

Background: Patients with acute venous thromboembolism (VTE) need anticoagulation (AC) therapy for at least 3/6 months (primary treatment); after that period, they should receive a decision on the duration of therapy. Methods: This study examined the complications occurring during two years of follow-up (FU) in patients with a first VTE who were recruited in 20 clinical centers and had discontinued or prolonged AC. They were included in the START2-POST-VTE prospective observational study. Results: A total of 720 patients (53.5% males) who, after the completion of primary treatment, had received the decision to continue (*n* = 281, 39%; 76.1% with a DOAC) or discontinue (*n* = 439, 61%) AC were followed up for 2 years (total FU = 1318 years). The decision to prolong or suspend AC was made in similar proportions in patients with unprovoked or provoked index events. Courses of sulodexide treatment or Aspirin (100 mg daily) were prescribed to 20.3% and 4.5%, respectively, of the patients who discontinued AC. The bleeding rate was significantly higher in patients who extended AC (1.6% pt/y) than in those who stopped AC (0.1% pt/y; *p* = 0.001) and was higher in patients using standard-dose DOACs (3.1% pt/y) than in those using reduced-dose DOACs (0.4% pt/y). The recurrent VTE rates were similar between the two groups (2.2% pt/y during AC vs. 3% pt/y off AC). Conclusion: Physicians’ decisions about AC duration were independent of the unprovoked/provoked nature of the index event. The bleeding rate was higher in patients who continued AC using standard-dose DOACs. Surprisingly, the rate of thrombotic recurrence was not different between those who continued or discontinued AC. Randomized studies comparing different procedures to decide on the duration of AC after a first VTE are needed.

## 1. Introduction

Venous thromboembolism (VTE), which includes deep vein thrombosis (DVT) and/or pulmonary embolism (PE), is a common and potentially serious disease that urgently requires active anticoagulant treatment. International guidelines unanimously recommend that all patients with acute VTE should receive full-dose anticoagulation for at least 3–6 months to complete the primary treatment [1,2,3,4]. However, the disease is subject to recurrence if anticoagulant treatment is stopped. The risk of recurrence is not uniform among patients; it is very high in those with persistent and strong risk factors (such as cancer), high in those with unprovoked events, low when the event is associated with transient and weak risk factors, and very low when it occurs in association with major surgery or other serious clinical conditions [5]. According to a recent systematic review and meta-analysis that included 18 studies and 7515 patients with unprovoked VTE [6], the recurrence rate one year after anticoagulant withdrawal was 10.3%, with a cumulative incidence at 5 years of 25%. These data seem to support prolonged anticoagulation beyond the first 3–6 months in patients with unprovoked VTE, provided that they are not at high risk of bleeding. However, the risk of bleeding during prolonged anticoagulation is not easy to predict, and most currently available predictive clinical scores have proven unsatisfactory [7]. In addition, the dichotomy between unprovoked and provoked VTE events when assessing the risk of recurrence has been questioned by some authors [8], who suggested considering all risk factors for recurrence, which are multiple and vary in nature and type. In this context, a recent analysis of studies on the use of rivaroxaban for extended treatment showed that recurrence rates in patients with VTE associated with minor persistent or minor transient risk factors were not significantly lower than those in patients with unprovoked VTE, demonstrating that prolonged treatment is beneficial in patients with both unprovoked and provoked VTE events [9]. For all of these reasons, guidelines and recommendations are difficult to translate into clinical practice, and the decision on anticoagulation duration is influenced by many factors, such as the physician’s experience, confidence in guidelines and recommendations, and patient characteristics and preferences. In this regard, recent reports on the management of patients with VTE from a number of countries point to the wide variety of practices adopted by different physicians [10,11] and clinical centers [12,13].

In a recent publication [12], we presented data on decision making for treatment beyond the initial and maintenance therapy phases from Italian treatment centers (mainly vascular centers) in VTE patients enrolled in the Italian prospective START2-POST-VTE observational study. Nearly 60% of the patients who had completed the maintenance treatment phase at the time of publication continued some form of treatment (mainly with anticoagulants), while the remaining patients discontinued all treatment. The decision to extend or discontinue anticoagulation was made in a similar proportion of patients whether the index event was unprovoked or provoked. 

The main objective of the present report was to analyze the occurrence of complications during two years of follow-up in patients currently present in the START2-POST-VTE study who discontinued or prolonged anticoagulation.

## 2. Methods

The START2-Register (Survey on anticoagulated pAtients RegisTer, NCT02219984), which is promoted and funded by the “Arianna Anticoagulazione” Foundation based in Bologna, Italy, is an ongoing multicenter, prospective, observational study that has been described in detail elsewhere [14]. START2 POST-VTE, a part of the START2-Register, includes patients with a recent VTE episode who have given their written informed consent. The Foundation has received an unrestricted financial grant from Alfasigma (Bologna, Italy) to support the conduct of the START2 POST-VTE registry. Fifteen clinical centers (9 thrombosis centers affiliated with the Italian Federation of Anticoagulation Clinics, 4 centers operating in angiology departments, and 2 centers in internal medicine departments) and 1 vascular specialist participated in the START2-POST-VTE study. All participating physicians were expert vascular specialists. 

Patients who were aged ≥18 years were enrolled in the study during primary treatment following diagnosis of a first-ever DVT and/or PE event. Patients were eligible if they were treated with anticoagulant therapy for 3–24 months following the VTE event. Subjects provided written informed consent prior to inclusion in the study. The exclusion criteria were an age of <18 years, thrombosis in other sites, inability or unwillingness to provide written informed consent, and the presence of other diseases requiring anticoagulation. Each subject had the right to withdraw from the study at any time and without giving a reason. All decisions regarding the type, dose, and duration of patient treatment before and after enrollment in the study were left to the discretion of the treating physician, who was required to report (a) when the patient was evaluated after the index event to determine the duration of anticoagulation, (b) what the decision was, and (c) the reasons for the decision. The participating physicians were also asked to follow up their patients for at least 6 months and, hopefully, for two years after the decision. All of the investigators recorded patient information in a structured case report form (CRF) in a web-based central electronic database. The information collected was checked by a dedicated study monitor from the Foundation (E.A.). All patients were assigned a unique identifier, and the data were anonymized prior to their registration in the CRF. 

Patient information was collected electronically in the registry’s central database. Patient enrollment began in April 2017 and ended in December 2019. At the time of patient enrollment, participating physicians were asked to collect demographic and clinical characteristics, risk factors for bleeding and thrombotic complications, routine laboratory data, the type, site, and clinical aspects of the index VTE episode, the time of its occurrence, the type of anticoagulant therapy used, and the presence of concomitant medication with antiplatelet drugs. Laboratory tests were not mandatory and were performed by local hospital laboratories. Creatinine clearance was calculated according to the Cockcroft–Gault formula [15], and renal failure was defined according to the National Kidney Foundation stratification [16]. We also calculated Charlson’s weighted comorbidity index score, which combined both age and comorbidity [17], and we decided to stratify the weighted comorbidity index into 3 classes: mild for patients with a score of 0 to 1; moderate for patients with a score of 2 to 4; severe for patients with a score higher than or equal to 5.

### 2.1. Follow-Up Data

For the purposes of the present analysis, follow-up was considered to extend from the time at which the participating physicians made a treatment decision to (1) the completion of two years of follow-up, (2) until December 2021, (3) until the last available follow-up for patients who were subsequently lost to follow-up or declined further participation in the START2-Register, or 4) until the occurrence of a disease requiring prolonged anticoagulation, major bleeding (MB), thrombotic complications, or death—whichever occurred first. MB was defined according to the International Society on Thrombosis and Hemostasis criteria [18]. Clinically relevant non-MB (CRNMB) events, which were defined as any overt bleeding requiring medical intervention and/or treatment discontinuation that did not meet any of the criteria for MB, were also recorded [19]. Thromboembolic events were recorded and defined as clinically confirmed recurrent VTE episodes, venous thrombosis at different sites, superficial vein thrombosis, stroke/arterial thromboembolism/transient cerebral ischemic attack, or myocardial infarction. Detailed clinical reports of any relevant clinical event that occurred during follow-up were also collected.

### 2.2. Statistical Analysis

Continuous variables are expressed as the median with interquartile range (IQR) or as the mean plus or minus standard deviation (SD). Categorical variables are expressed as frequencies and percentages. Differences between continuous variables were assessed using an unpaired t-test, and categorical variables were compared by using the Chi-square test or Fisher exact test, as appropriate. The median and interquartile range (IQR) of the follow-up time was calculated, and the median test applied to score differences between groups is reported. The incidence of recurrence, death, and bleeding events was calculated by dividing the number of events by a person’s time at risk. The incidence ratio was calculated together with the 95% confidence interval (95% CI). Univariate logistic regression analysis was performed to explore the association between the clinical condition and the decision to extend anticoagulation. All variables were subsequently entered into a multivariable analysis, and multiple logistic regression with backward selection was performed to identify the factors that were independently associated with the decision to extend anticoagulation. The results are presented as an odds ratio (OR) with the 95% confidence interval (CI). *p* < 0.05 was considered statistically significant. The data were analyzed with the use of the SPSS software for Windows, V.26 (SPSS Statistic, IBM, Armonk, NY, USA), and the Stata V.14 statistical software package (Stata Corp, College Station, TX, USA).

## 3. Results

### 3.1. Patients

A total of 720 patients (53.5% male) were included in the study; their characteristics, the type and site of venous thrombotic events, and the anticoagulant treatments received before inclusion are shown in Table 1. After a single VTE event, all patients received anticoagulation therapy for initial and primary treatment for a mean duration of 8.7 months. The index events were classified as unprovoked in 56.9% of cases and as provoked in the remaining cases. The site of the index event was proximal DVT in 55.0% of patients. After completing primary treatment, which consisted of anticoagulation with a DOAC in 81.5% of cases, all patients were assessed by their treating physicians, who recommended the discontinuation (439 subjects, 61%) or extension (281, 39%) of anticoagulation. As shown in Table 2, the decision to extend or discontinue anticoagulation did not prevalently depend on the unprovoked or provoked nature of the index events. The site of the events was more important for that decision. In fact, prolongation of anticoagulation was preferred in cases with proximal DVT (62.2%), whereas anticoagulation was definitively discontinued in most cases with isolated distal DVT (82.2%). The presence of thrombophilic abnormalities was also a factor favoring prolongation of treatment. The most frequent reasons for treating physicians’ decision to extend anticoagulation were the patient’s general condition being considered to be at high risk of recurrence, recently assessed high D-dimer levels, persistent residual vein thrombosis, and the presence of thrombophilic alterations. In the multivariable logistic analysis (Table 3), the factors most associated with the extension of anticoagulation were the unprovoked nature of the index event, the presence of thrombophilic alterations, a proximal DVT or an isolated distal DVT (associated with discontinuation) as the index event, and an overall assessment of being at high risk of VTE recurrence. 

### 3.2. Follow-Up

The total follow-up duration was 1318 years. No patients withdrew from the study. Most of the patients who continued anticoagulation (*n* = 281) were treated with a DOAC during follow-up at either the standard (45.5%) or a reduced dose (46.3%); very few patients received VKAs (5.0%) or LMWH (3.2%) (Table 4). Among the patients who discontinued anticoagulation (*n* = 439), 30 (4.5%) were prescribed aspirin (100 mg daily), while 89 (20.3%) were recommended cycles of sulodexide.

**Figure 1:**
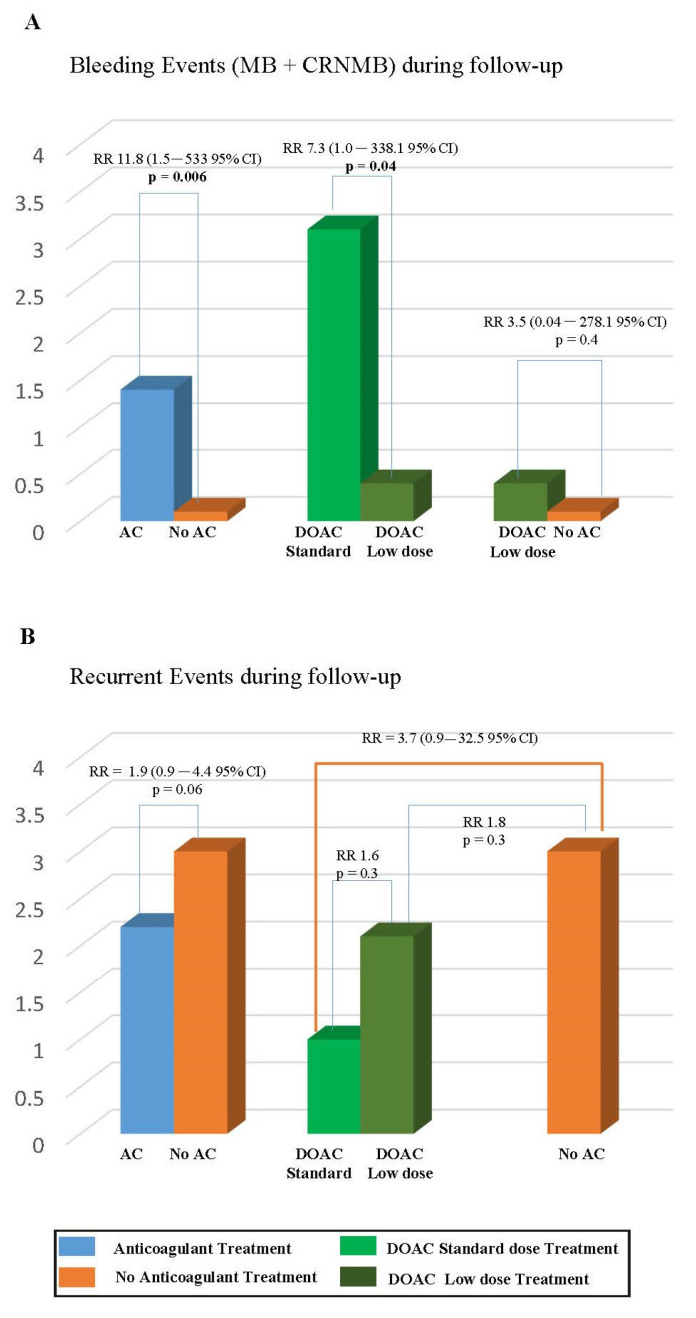
Adverse events (**A**) bleeding events; (**B**) recurrent events in patients who continued or discontinued anticoagulation.

Table 5 and Figure 1 show the number and incidence of primary outcomes occurring during follow-up in the 720 patients enrolled in the study. More bleeding events (MB+CRNMB) occurred in patients who prolonged anticoagulation (1.6% pt/y) than in those who discontinued anticoagulation (0.1% pt/y, *p* = 0.001). In the latter group, only one CRNMB occurred in a patient receiving aspirin. Bleeding was also more common in patients treated with a standard-dose DOAC (3.1% pt/y) than in those on reduced-dose DOACs (0.4% pt/y, *p* = 0.028), whereas no significant differences in the incidence of bleeding were observed between patients treated on reduced-dose DOACs and those who discontinued anticoagulation (*p* = 0.320) (Figure 1, Panel A).

The incidence of recurrent venous thrombotic events was not different between patients who prolonged anticoagulation (2.2% pt/y) and those who discontinued anticoagulation (3.0% pt/y, RR 0.56; 0.24–1.2 95% CI; *p* 0.1); similarly, no differences were detected in the incidence of arterial events and of all-cause death between the two groups (Table 5) (no deaths could be attributed to thrombotic recurrence). Six superficial vein thromboses that were not considered to be thrombotic recurrences occurred in patients who discontinued anticoagulation. 

## 4. Discussion

In this study, we analyzed the long-term outcomes (bleeding or recurrent events and death) during two years of follow-up in a cohort of 720 patients in whom the treating physicians had prescribed prolongation or discontinuation of anticoagulation after a first venous thromboembolism (DVT and/or PE). Prior to enrollment, all patients had received anticoagulation therapy as initial and primary treatment for an average of 8.7 months. The clinically relevant findings of this study are as follows. First, while the incidence of bleeding events (including MB and CRNMB) was generally higher in those who continued anticoagulation, this was true for patients receiving standard doses but not for those treated with reduced-dose DOACs (in 88% of patients, it was apixaban 2.5 mg twice daily), where the incidence of bleeding was not significantly higher than that in patients who discontinued anticoagulation. This result confirmed the high safety of low-dose DOACs shown in available trials [20,21], as well as in real-life studies [22,23]. Second, the incidence of thrombotic recurrence during follow-up did not differ between patients who continued anticoagulation (with a DOAC in 92% of cases) and those who discontinued anticoagulation. This is an important and unexpected finding of our study. This finding contrasts with the results of two intervention trials on the use of reduced-dose DOACs [20,21] and a recent real-life study using apixaban or warfarin for extended anticoagulation [24]. Furthermore, our results seem to raise doubts about the benefits of extending anticoagulation beyond primary treatment. These findings, which differ from those of the available studies, are not easy to explain. The present study was a real-life study in which the participating physicians decided whether to extend anticoagulation after assessing the patients’ characteristics. We speculate that they were able to differentiate between patients at low and high risk of recurrence and, thus, to give extended treatment only to the latter. According to this reasoning, extended anticoagulation was effective in reducing the risk of recurrence in these patients to a level similar, if not lower, than that in patients in whom anticoagulation was discontinued because they were considered to be at low risk. The two intervention trials (Amplify Extension and Einstein Choice) showed that both standard and reduced doses were effective in reducing the risk of recurrence compared to placebo and aspirin, respectively. However, both trials, as well as the aforementioned real-life study [24], included patients who were in equipoise for continued anticoagulation. Hence, we believe that the different study designs may explain our different results.

In the present study, the treating physicians did not decide on the duration of anticoagulant treatment primarily based on the nature of the index event—unprovoked or provoked—but rather on a thorough and complete evaluation of patient characteristics. Our results are fully in line with those of a recent prospective international study [22] and seem to support the thesis of those who, in contrast to international guidelines and recommendations [1], caution against the use of unprovoked/provoked events as the sole VTE marker for deciding on the extension of anticoagulation [8] and instead strive for a more complex risk stratification of patients.

Our study has some limitations that should be mentioned. With this real-life observational study, we aimed to evaluate (a) the decisions of treating physicians regarding the extension or discontinuation of anticoagulation in patients after a previous single VTE event and (b) the clinical consequences of such decisions. For these reasons, the participating physicians were not given any criteria to guide their decisions, and they were left entirely to their discretion. The number of patients included in the study was substantial; however, the enrollment of more patients would likely have increased the probability that some differences in outcomes would have reached statistical significance (e.g., the incidence of recurrent events in patients receiving standard-dose DOACs versus patients not on anticoagulation). Although it is well known that some drugs (e.g., amiodarone, dronedarone, and verapamil, which are particularly used in atrial fibrillation patients, who were excluded from this study) may increase the DOAC levels and the risk of bleeding, we admit that the treatment with these drugs at baseline or during follow-up was not collected in CRF, as was also the case for some laboratory results. Finally, there was no central adjudication of outcome events in this study. The strengths of our study include its prospective design, large cohort, and long follow-up. 

In conclusion, patients with a prior single VTE event who had completed approximately 8 months of anticoagulation as a primary treatment were included in the study after a decision by their treating physicians, which was not primarily driven by the provoked or unprovoked nature of the index events, to extend (in 39% of patients, almost all of whom received a DOAC) or discontinue (62%) anticoagulation and were followed up for two years. At the end of the two years, the recurrence of thrombotic events was similar in patients who either continued or discontinued anticoagulation. In contrast, patients who continued anticoagulation had a low incidence of MB+CRNMB (1.6% pt/y), which was, however, significantly higher than that recorded in patients who discontinued anticoagulation. A higher incidence of bleeding was only observed in patients treated with the standard doses of DOACs. The results of our study seem to cast doubt on the wisdom of prolonging anticoagulation in all patients with unprovoked index events, as recommended by some international guidelines [1], and even more so in patients with provoked events, as some authors suggested [9]. Our results seem to indicate that, in general, the treating physicians were able to discriminate between patients at high versus low risk of recurrence using a global evaluation of patient characteristics; this approach was effective and safe for long-term clinical outcomes. We believe that it is time to compare different approaches (rather than only the criterion of being provoked/unprovoked) to deciding on the duration of anticoagulation by promoting large randomized clinical trials. 

## Figures and Tables

**Table 1:** Characteristics of patients, index events, and anticoagulant treatments.

	All Patients
**Number**	720
Males, *n* (%)	385 (53.5)
Age, mean (SD) years	65 (15)
Duration of primary treatment, months, mean ± SD	8.7 ± 6.5
**Nature of index event, *n* (%)** Unprovoked	410 (56.9)
**Site of index event, *n* (%)** Proximal DVT DVT+PE Isolated PE Isolated Distal DVT	398 (55.0) 76 (10.6) 99 (13.8) 146 (20)
**Type of anticoagulant treatment, *n* (%)** VKA LMWH/Fondaparinux DOACs Apixaban Dabigatran Edoxaban Rivaroxaban	60 (8.3) 73 (10.1) 587 (81.5) 158 55 108 266
Concomitant antiplatelet treatment, *n* (%)	77 (10.6)

SD: standard deviation, DVT: deep vein thrombosis, VKA: vitamin K antagonist, LMWH: low-molecular-weight heparin, DOACs: direct oral anticoagulants.

**Table 2:** Characteristics of patients in whom anticoagulant treatment was extended or discontinued.

	Anticoagulation Extended	Anticoagulation Discontinued	*p*
Number, (%)	281 (39.0)	439 (61.0)	
Male Sex,	160 (57.0)	225 (51.2)	0.128
Age, mean (SD) years	68 (14)	63 (16)	0.034
Duration of primary treatment, months; m ± SD	10.8±8.0	7.3± 6.2	0.001
**Nature of index event:** Unprovoked, *n* (%) Provoked	167 (59.4) 114 (40.5)	243 (55.3) 196 (44.6)	0.315 0.278
**Site of index event** Proximal DVT DVT + PE Isolated PE Isolated Distal DVT	175 (62.2) 38 (13.5) 42 (14.9) 26 (9.2)	223 (55.3) 38 (8.6) 57 (13.0) 120 (27.3)	0.067 0.036 0.470 0.0001
**Type of anticoagulant used during primary treatment** VKA LMWH/Fondaparinux DOACs Apixaban Dabigatran Edoxaban Rivaroxaban	35 (12.5) 32 (11.4) 214 (76.1) 72 9 44 89	25 (5.7) 41 (9.3) 373 (85.0) 86 46 64 177	0.002 0.362 0.003
Concomitant antiplatelet treatment	24 (8.5)	30 (6.8)	0.240
**Comorbidity** None Previous TIA/stroke episode Previous major bleeding episode Hypertension (drug treatment) Diabetes mellitus Ischemic heart/peripheral diseases Heart failure Chronic inflammatory diseases Active cancer Glomerular Filtration Rate [ml/min, mean (SD)] Severe renal disease (>30 mL/min) Moderate renal disease (30–60 mL/min) Thrombophilia abnormalities Charlson’s index 0–1 (mild) 2–4 (moderate) ≥5 severe	87 (30.3) 10 (3.6) 5 (1.8) 114 (40.6) 21 (7.5) 12/7 (6.7) 9 (3.2) 16 (5.7) 22 (7.8) 80 (59;105) 2 (0.7) 68 (24.2) 48 (17.1) 47 (16.7) 142 (50.5) 92 (32.7)	146 (33.2) 11 (2.5) 10 (2.3) 168 (38.3) 45 (10.2) 28 (6.4) 8 (1.8) 17 (3.9) 20 (4.5) 88 (37) 5 (1.1) 94 (21.4) 37 (8.4) 77 (17.5) 247 (56.2) 115 (26.1)	0.567 0.393 0.649 0.538 0.220 0.874 0.226 0.261 0.064 0.712 0.588 0.380 0.0004 0.839 0.145 0.063

DOAC: direct oral anticoagulant, DVT: deep venous thrombosis, LMWH: low-molecular-weight heparin, PE: pulmonary embolism, TIA: transient ischemic attack, VKA: vitamin K antagonist.

**Table 3:** Factors associated with the extension of anticoagulation in the univariate and multivariate analyses.

	Univariate Analysis			Multivariable Analysis		
Factors	OR	95% CI	*p*	OR	95% CI	*p*
Age *	1.0	1.0–1.02	0.03	2.2	0.85–5.9	0.1
Thrombophilia abnormalities	2.2	1.4–3.5	0.01	2.2	1.5–3.8	0.01
Unprovoked Event	1.8	1.3–2.4	0.001	1.7	1.2–1.8	0.001
Proximal DVT	2.2	1.3–4.2	0.02	1.8	1.1–3.9	0.03
Distal DVT	0.7	0.58–0.79	0.01	0.2	0.72–0.82	0.02
Isolated PE	2.1	1.5–7.1	0.02	2.1	0.91–7.8	0.06
Charlson’s score (moderate) °	1.1	0.7–1.9	0.7	1.1	0.58–5.3	0.3
High risk for VTE recurrence #	2.3	1.3–4.2	0.01	2.2	1.1–4.6	0.02
Antiplatelet therapy	0.8	0.5–1.5	0.6	0.5	0.4–1.6	0.6

* Age is considered as a continuous variable. ° Charlson’s score: mild class vs. moderate/high classes. # Individual thrombotic risk assessment of patients formulated by the treating physicians. DVT: deep venous thrombosis, PE: pulmonary embolism, VTE: venous thromboembolism.

**Table 4:** Treatments received during follow-up.

Treatments	Patients, *n* (%)	FU (Years)
All Patients, *n*. (%)	720	1318
Patients who continued anticoagulation ($)	281 (39.0)	490
DOACs (standard dose) Apixaban Dabigatran Edoxaban Rivaroxaban	128 (45.5) 23 6 30 69	190.2
DOACs (reduced dose) Apixaban (2.5 mg BID) Rivaroxaban (10 mg OID)	130 (46.3) 114 16	233.5
VKA	14 (5.0)	35.2
LMWH/Fondaparinux	9 (3.2)	32.3
Patients who discontinued anticoagulation	439 (61.0)	828
No treatment at all Sulodexide (&) Aspirin	330 (75.2) 89 (20.3) 20 (4.5)	615.0 173.4 39.4

FU: follow-up, DOACs: direct oral anticoagulants, VKA: vitamin K antagonist, LMWH: low-molecular-weight heparin. ($): One patient continued anticoagulation for the presence of antiphospholipid syndrome, and four did so for atrial fibrillation occurrence. These patients were censored at the moment these diseases were reported by the participant centers. (&): Unlike anticoagulant drugs and low-dose aspirin (100 mg per day), Sulodexide is not reimbursed by the National Health System in Italy; in Italy, the drug is usually prescribed at the dose of one 250-lipasemic-unit capsule twice daily for cycles of treatment of one month, 2 or 3 times per year.

**Table 5:** Adverse events that occurred during follow-up.

Patients	*n*. (FU, Years)	Bleeding (Including Major and CRNMB) *n* (×100 pt-y)	Recurrent VTE (Including Proximal/Distal DVT and PE) *n* (×100 pt-y)	Arterial Events (Including AMI, TIA/Stroke *n* (×100 pt/y)	Death *n* (%)
All	720 (1318)				35 (4.9)
**Anticoagulation continued** **All patients** DOAC (all doses) Standard dose Reduced dose VKA LMWH	**281 (490)** 258 (424) 128 (191) 130 (233) 14 (35) 9 (32)	**7 (1.4)** 7 (1.6) 6 (3.1) 1 (0.4) 0 0	**10 (2.2)** 7 (1.6) 2 (1.0) 5 (2.1) 2 (5.7) 1 (3.1)	**2 (0.4)** 2 (0.5) 1 (0.5) 1 (0.4) 0 0	**9 (3.2)** 8 (3.1) 1 (0.8) 7 (5.3) 0 1 (3.1)
**Anticoagulation discontinued** **All patients** No specific treatment Sulodexide (&) Aspirin	**439 (828)** 330 (615) 89 (173) 20 (39)	**1 (0.1)** 0 0 1 (2.6)	**33 (3.0)** 23 (3.7) 7 (4.0) 3 (7.7)	**4 (0.5)** 2 (0.3) 2 (1.2) 0	**26 (5.9)** 21 (6.4) 3 (3.3) 2 (10.0)
**RR for total mortality** AC continued AC discontinued	9/490 1.8 ×100 pt/y 26/828 3.1 ×100 pt/y RR 1.7 (95% CI 0.77–4.1) *p* = 0.1				

(&): Usually prescribed at the dose of one 250-lipasemic-unit capsule twice daily for cycles of treatment of one month, 2 or 3 times per year. AMI = acute myocardial infarction, FU = follow-up, CRNMB = clinically relevant non-major bleeding, DOAC = direct oral anticoagulant, LMWH = low-molecular-weight heparin, VKA = vitamin K antagonist.

## Data Availability

The data presented in this study are available upon reasonable request from Gualtiero Palareti.
